# Genome-wide identification of the class III peroxidase gene family of sugarcane and its expression profiles under stresses

**DOI:** 10.3389/fpls.2023.1101665

**Published:** 2023-01-30

**Authors:** Heyang Shang, Linqi Fang, Lifang Qin, Hongtao Jiang, Zhenzhen Duan, Hai Zhang, Zongtao Yang, Guangyuan Cheng, Yixue Bao, Jingsheng Xu, Wei Yao, Muqing Zhang

**Affiliations:** ^1^National Engineering Research Center for Sugarcane & Guangxi Key Laboratory of Sugarcane Biology, Fujian Agriculture and Forestry University, Fuzhou, China; ^2^State Key Laboratory for Conservation and Utilization of Subtropical Agro-Bioresources & Guangxi Key Laboratory of Sugarcane Biology, Guangxi University, Nanning, China

**Keywords:** sugarcane, class III peroxidase, sugarcane mosaic virus, cadmium, salt stress

## Abstract

**Introduction:**

Plant-specific Class III peroxidases (PRXs) play a crucial role in lignification, cell elongation, seed germination, and biotic and abiotic stresses.

**Methods:**

The class III peroxidase gene family in sugarcane were identified by bioinformatics methods and realtime fluorescence quantitative PCR.

**Results:**

Eighty-two PRX proteins were characterized with a conserved PRX domain as members of the class III PRX gene family in R570 STP. The ShPRX family genes were divided into six groups by the phylogenetic analysis of sugarcane, Saccharum spontaneum, sorghum, rice, and *Arabidopsis thaliana*. The analysis of promoter *cis*-acting elements revealed that most *ShPRX* family genes contained *cis*-acting regulatory elements involved in ABA, MeJA, light responsiveness, anaerobic induction, and drought inducibility. An evolutionary analysis indicated that ShPRXs was formed after *Poaceae* and *Bromeliaceae* diverged, and tandem duplication events played a critical role in the expansion of *ShPRX* genes of sugarcane. Purifying selection maintained the function of *ShPRX* proteins. *SsPRX* genes were differentially expressed in stems and leaves at different growth stages in *S. spontaneum*. However, *ShPRX* genes were differentially expressed in the SCMV-inoculated sugarcane plants. A qRT-PCR analysis showed that SCMV, Cd, and salt could specifically induce the expression of PRX genes of sugarcane.

**Discussion:**

These results help elucidate the structure, evolution, and functions of the class III *PRX* gene family in sugarcane and provide ideas for the phytoremediation of Cd-contaminated soil and breeding new sugarcane varieties resistant to sugarcane mosaic disease, salt, and Cd stresses.

## 1 Introduction

Sugarcane, one of the critical sugar and energy crops, is often subjected to various biotic stresses, including *Sugarcane mosaic virus* (SCMV), *Sorghum mosaic virus* (SrMV), *Sugarcane streak mosaic virus* (SCSMV), and abiotic stress, including salt, heavy metal, and drought stress. Sugarcane mosaic disease, caused by SCMV, SrMV, and SCSMV, is currently one of the most severe sugarcane diseases worldwide that adversely affect the healthy and sustainable development of the sugarcane industry (Yao et al., 2017; Moradi et al., 2018; Rice et al., 2019; Rice and Hoy, 2020). Under salt stress, the growth of sugarcane is hindered, seriously affecting the quality of sugarcane and even causing a large area of yield reduction or crop failure. Soil heavy metal pollution, one of the leading environmental stresses affecting plant growth and development, is becoming the prime concern of various terrestrial ecosystems worldwide. Among heavy metals, cadmium (Cd), one of the most dangerous toxic elements for plants, inhibits various physiological processes of plants, including seed germination, seedling growth, photosynthesis, and antioxidant system (Zhu et al., 2021). The contents of chlorophyll and soluble protein decreased significantly, whereas the content of carotenoids increased significantly in the Cd-treated sugarcane. The activity of ascorbate peroxidase, peroxidase (PRX), and catalase (CAT) increased significantly (Yousefi et al., 2018). Sugarcane, one of the cultivated crops with the highest biomass and solid tolerance to Cd, can be used as a candidate crop for the phytoremediation of Cd pollution in soil (Sereno et al., 2007; Yousefi et al., 2018). Therefore, it is one of the main challenges to improve the yield and quality of sugarcane and the ability to repair Cd pollution in soil by improving the resistance of sugarcane to biotic and abiotic stresses.

The PRXs, the critical enzymes of peroxisomes, widely exist in animals, plants, and microorganisms. According to the protein structural and functional characteristics, PRXs are divided into heme PRXs and non-heme PRXs. Heme PRXs are further subdivided into animal PRXs and non-animal PRXs. According to the sequence and catalytic characteristics of proteins, non-animal heme PRXs comprise Class I, II, and III PRXs, all containing a heme group consisting of protoporphyrin IX and iron (III). Class I PRXs widely exist in most organisms, such as plants, fungi, bacteria, and protozoa. However, Class II PRXs exist in fungi, and class III PRXs only exist in plants (Piontek et al., 2001; Zamocky, 2004; Zamocky et al., 2010; Shigeto and Tsutsumi, 2016). Class III PRXs are classical plant secretory PRXs that play a crucial role in lignification, cell elongation, and seed germination (Lee, 1977; Piontek et al., 2001; Shigeto and Tsutsumi, 2016; Kidwai et al., 2020). Numerous class III PRXs were present in the cell walls, and the balance of cell wall loosening and stiffening could be precisely controlled by the antagonistic activities of class III PRXs during plant growth (Francoz et al., 2015). Class III PRXs was identified as an essential enzyme for lignin biosynthesis in plants. Coniferyl alcohol and sinapyl alcohol, precursors for the synthesis of lignin monomers, could be catalyzed by the class III PRX gene *PbPRX2* in Chinese pear fruit (Vanholme et al., 2019; Zhu et al., 2021). In addition, class III PRXs were involved in the internal browning of pineapple (Hou et al., 2022) and closely related to pollen fertility in *Gossypium hirsutum* (Chen et al., 2022).

The class III PRX genes play a vital role in responses to various biotic and abiotic stresses throughout the plant life cycle. The wheat class III PRX gene *(TaPRX-2A)* increased the activities of superoxide dismutase (SOD), PRX, and CAT to scavenge reactive oxygen species (ROS) (Su et al., 2020). In sweet potato, the B-box family transcription factor *IbBBX24* activated the expression of the class III PRX gene *IbPRX17* by binding to the promoter of *IbPRX17*, and the overexpression of *IbPRX17* significantly improved the tolerance to salt and drought stresses by scavenging ROS (Zhang et al., 2022). Overexpressed the class III PRX gene *(OsPRX38*) in *Arabidopsis thaliana* exposed to arsenic stress increased SOD, PRX, and GST activities to reduce the content of hydrogen peroxide (H_2_O_2_), electrolyte leakage, and malondialdehyde (Kidwai et al., 2019). In rice, the overexpression of the class III PRX *OsPRX30*, maintaining a high level of PRX activity and reducing the content of H_2_O_2_, reduced bacterial blight resistance (Liu et al., 2021). In *Citrus sinensis*, the class III *CsPRX* family genes, induced by salicylic acid and methyl jasmonate, were involved in citrus bacterial canker disease (Li et al., 2020).

The publication of the genome of *Saccharum* hybrid cultivar R570 (R570) BAC clone (BAC) and single tiling path (STP) has necessitated the study of the function of the sugarcane class III PRX gene. At present, the class III PRX gene family has been studied in various plant species, including *A. thaliana* (Tognolli et al., 2002), rice (Passardi et al., 2004), maize (Wang et al., 2015), potato (Yang et al., 2020), soybean (Aleem et al., 2022), *Brachypodium distachyon* (Zhu et al., 2019), and allotetraploid cotton (Duan et al., 2019). However, it has yet to be reported on the identification and characterization of the class III PRX gene family in sugarcane.

A genome-wide search was carried out on class III PRXs in R570 STP, R570 BAC, *Saccharum spontaneum* AP85-441 (*S. spontaneum* AP85-441), sorghum, *A. thaliana*, and rice. The functions of the class III PRX genes in sugarcane were analyzed concerning the gene structure, conserved motif, *cis*-acting elements, codon usage bias, and evolutionary analysis. The expression level of *ShPRXs* was studied under SCMV, Cd, and salt stress response. The results from this study help elucidate the structure, evolution, and functions of the class III PRX gene family.

## 2 Materials and methods

### 2.1 Identification of PRX family members

Genome data of R570 BAC and R570 STP were obtained from the sugarcane genome hub (https://sugarcane-genome.cirad.fr/; Garsmeur et al., 2018). Genome data of *A. thaliana* TAIR10, *Oryza sativa* (IRGSP-1.0), *S. bicolor* (NCBIv3), and *S. spontaneum* AP85-441 were obtained from the Ensembl Plants database (http://plants.ensembl.org/index.html; Yates et al., 2022).

The hidden Markov model (HMM) file of the PRX domain (PF00141) was downloaded from the Pfam database (https://pfam-legacy.xfam.org/; Sonnhammer et al., 1998; Blom et al., 1999; Mistry et al., 2021). The HMMER software (version 3.1b1; http://www.hmmer.org/) was used to search against the whole genome protein file of R570, *A. thaliana*, *O. sativa*, *S. bicolor*, and *S. spontaneum* under the condition of e-value < 1×10^−20^. Multiple sequence alignment was performed using ClustalW (http://www.clustal.org/), and a new HMM matrix file was constructed. The PRX domain-containing proteins were searched on the new HMM matrix file and screened by e-value < 0.001. The PRX protein Ref-seq of all plants was downloaded to build a library from the NCBI database (https://www.ncbi.nlm.nih.gov/). The putative PRX genes were searched using BLASTP and screened by e-value < 1×10^−10^ and identity > 75%. The protein-conserved domains were verified by Pfam and NCBI CDD (https://www.ncbi.nlm.nih.gov/Structure/bwrpsb/bwrpsb.cgi), and the genes without the PRX domain were deleted (Lu et al., 2020; Mistry et al., 2021). In addition, the PRX gene family was identified based on the sugarcane transcriptome data.

The physicochemical properties of the *ShPRX* family proteins were calculated using ExPASy-ProtParam (https://web.expasy.org/protparam/; Wilkins et al., 1999). The subcellular localization of the *ShPRX* family proteins was predicted using Plant-mPLoc (http://www.csbio.sjtu.edu.cn/bioinf/plant-multi/; Chou and Shen, 2010). The transmembrane domain of the *ShPRX* family proteins was predicted using TMHMM 2.0 (https://services.healthtech.dtu.dk/service.php?TMHMM-2.0; Sonnhammer et al., 1998). The signal peptide of the *ShPRX* family proteins was predicted using SignalP 6.0 (https://services.healthtech.dtu.dk/service.php?SignalP; Teufel et al., 2022). The phosphorylation sites of the *ShPRX* family proteins were predicted using NetPhos 3.1 (https://services.healthtech.dtu.dk/service.php?NetPhos-3.1; Blom et al., 1999). The secondary structure of the *ShPRX* family proteins was analyzed using SOPMA (https://npsa-prabi.ibcp.fr/cgi-bin/npsa_automat.pl?page=npsa_sopma.html; Geourjon and Deleage, 1995).

### 2.2 Phylogenetic analysis

The multiple protein sequences of the PRX gene family in R570 STP, *A. thaliana*, *O. sativa*, *S. bicolor*, and *S. spontaneum* were compared using the MEGA-X MUSCLE software (https://www.megasoftware.net/; Kumar et al., 2018). The phylogenetic tree was constructed using the neighbor-joining method with the Jones–Taylor–Thornton model, 1,000 bootstrap replications, gamma distributed (G), and partial deletion gaps by MEGA-X. The phylogenetic tree was visualized using the online tool iTOL (https://itol.embl.de/; Letunic and Bork, 2021).

### 2.3 Chromosomal distribution, gene structure, and conserved motif analysis of the *ShPRXs*

The positional and structural information of *ShPRX* genes on the R570 STP chromosomes was extracted from the Generic Feature Format Version 3 (GFF3) (Garsmeur et al., 2018). The chromosomal locations of the *ShPRX* family genes were drawn using the online tool MG2C v2.1 (http://mg2c.iask.in/mg2c_v2.1/index.html). The conserved motifs of the *ShPRX* family proteins were identified using the online MEME with optimized parameters: minimum width, 6; maximum width, 50; and the number of motifs, 26 (Bailey and Elkan, 1994). The phylogenetic tree, gene structure, and conserved motifs of the *ShPRX* family genes were visualized using TBtools (Chen et al., 2020).

### 2.4 Analysis of *cis*-acting elements and codon usage bias of the *ShPRXs*

The sequence 2000 bp upstream from the start codon of *the ShPRX* family gene was obtained using the hybrid cultivar R570 reference genome (Garsmeur et al., 2018). The *cis*-acting elements of *ShPRX* family genes in promotor regions were predicted using the PlantCAR (Lescot et al., 2002). The phylogenetic tree and *cis*-acting elements of the *ShPRX* family genes were visualized using GSDS 2.0 (http://gsds.cbi.pku.edu.cn; Hu et al., 2015). The codon usage bias of the *ShPRX* family genes was analyzed using CodonW (https://codonw.sourceforge.net/) and EMBOSS: chips (https://www.bioinformatics.nl/cgi-bin/emboss/chips; Rice et al., 2000). The effective number of codon (ENc) plots and Parity rule 2 (PR2) plot analysis was performed as described by Wright (1990) and Chakraborty et al. (2020).

### 2.5 Syntenic and selection pressure analysis of the *PRX* family genes

The syntenic relationships of the PRX family genes were analyzed and visualized using MCScanX and Circos, respectively (Krzywinski et al., 2009; Wang et al., 2012). The frequencies of synonymous (Ks) and nonsynonymous (Ka) mutations, along with their ratios, were calculated to analyze the selection pressure of PRX duplicated gene pairs using TBtools (Chen et al., 2020). The divergence time (T) of class III PRX family genes was calculated as T = Ks/(2 × λ) × 10^−6^ Mya (λ = 6.5 × 10^−9^ for grasses; (Gaut et al., 1996). The divergence time values were estimated using TimeTree (http://www.timetree.org/; Kumar et al., 2022).

### 2.6 Expression profiles of the *PRX* family genes

The RNA-seq expression data of the growth and development of *S. spontaneum* were downloaded from the Saccharum Genome Database (SGD; http://sugarcane.zhangjisenlab.cn/sgd/html/index.html; Zhang et al., 2018). The transcriptome data of sugarcane were obtained by sequencing (Akbar et al., 2021). The gene expression data of rice under Cd (GSE35502) and salt (GSE60287) stresses were downloaded from the GEO database (https://www.ncbi.nlm.nih.gov/geo/; Ishimaru et al., 2012; Garg et al., 2015; Clough and Barrett, 2016; Shankar et al., 2016). The expression pattern of the PRX family genes was drawn based on FPKM values or log_2_ fold change (log_2_FC) using the R software (v4.0.5).

### 2.7 SCMV infection, Cd treatment, salt stress treatment, and qRT-PCR analysis

Badila and B48, grown to leaf stages 6–8 in the field, was inoculated using SCMV crude extract following Yao’s method (Yao et al., 2017), and the −3 and +1 leaves were collected at day 21 post-inoculation. The control plants were rubbed using 0.1 M phosphate buffer (pH 7.0). Sugarcane plants of Zhongzhe 1 were watered using 4.3-mM Cd solution or 0.5-L 1.0% sodium chloride solution when Zhongzhe 1 grew to leaf stage 3 in barrels containing 16 kilograms of soil. The control plants were watered using 0.5-L double-distilled water. The +1 leaves of sugarcane were collected at different time after treatment (0, 4, 8, 12, and 24 h).

Total RNA was extracted using Eastep^®^ Super Total RNA Extraction Kit (Promega (Beijing) Biotech Co. Ltd., Beijing, China) following the manufacturer’s instructions. The first strand of cDNA was synthesized using PrimeScript™ II 1^st^ Strand cDNA Synthesis Kit (Takara Biomedical Technology Co. Ltd., Beijing, China). The qRT-PCR experiment was conducted in three independent replicates using ChamQ Universal SYBR qPCR Master Mix (Nanjing Vazyme Biotech Co., Ltd., Nanjing, China) with the Bio-Rad CFX96 fluorescence quantitative PCR Instrument. The primers for qRT-PCR of 10 *PRX* genes were designed using Primer Premier 6. The relative expression levels of the PRX family genes were calculated using the 2^−ΔΔCt^ method, and statistical significance was analyzed using ordinary one-way ANOVA in GraphPad Prism 7.

## 3 Results

### 3.1 Identification and chromosomal distribution of the *PRX* gene family in sugarcane

Eighty-two PRX proteins with a conserved PRX domain were characterized as members of the class III *PRX* gene family in R570 STP and named *ShPRX1*–*ShPRX82* based on their respective locations on the chromosomes (**Tables S1** , **S2**). The total number of *PRX* family genes in the sugarcane R570 cultivar was lower than that in the *S. spontaneum* (113), sorghum (150), and rice (126), but slightly higher *in A. thaliana* (80) (**Table S2**).

The physical and chemical properties analysis revealed that the *ShPRX* family genes were predicted to encode polypeptides from 235 to 472 amino acids, with predicted molecular weights ranging from 25.11 to 49.76 kD. The theoretical pI ranged from 4.59 to 10.08, and the grand average of the hydropathicity values of 52 *ShPRX* proteins was negative, ranging from −0.51 to −0.002, indicating a hydrophilic characteristic. The grand average of the hydropathicity values of 30 *ShPRX* proteins was positive, ranging from 0.004 to 0.297, indicating a hydrophobic characteristic. The predicted number of negatively charged residues (Asp + Glu) in the *ShPRXs* was 14–59, and the number of positively charged residues (Arg + Lys) was 20–54. The instability index of 46 *ShPRX* proteins was less than 40, ranging from 25.38 to 39.76, indicating a stable characteristic. The instability index of 36 *ShPRX* proteins was greater than 40, ranging from 40.36 to 56.05, indicating an unstable characteristic (**Table S1**).

The subcellular localization prediction analysis showed that most *ShPRX* genes were located in the cytoplasm, and some were located in the vacuole, chloroplast, peroxisome, mitochondrion, cell membrane, and nucleus. Most *ShPRX* genes had signal peptides, 42 *ShPRX* genes had no transmembrane domain, and other *ShPRX* genes had only one transmembrane domain. The number of phosphorylation sites ranged from 16 to 47, while four *ShPRX* genes had no tyrosine phosphorylation site. The secondary structure prediction analysis showed that *ShPRX* proteins were mainly composed of alpha helix and random coil (**Table S1**).

**Figure 1:**
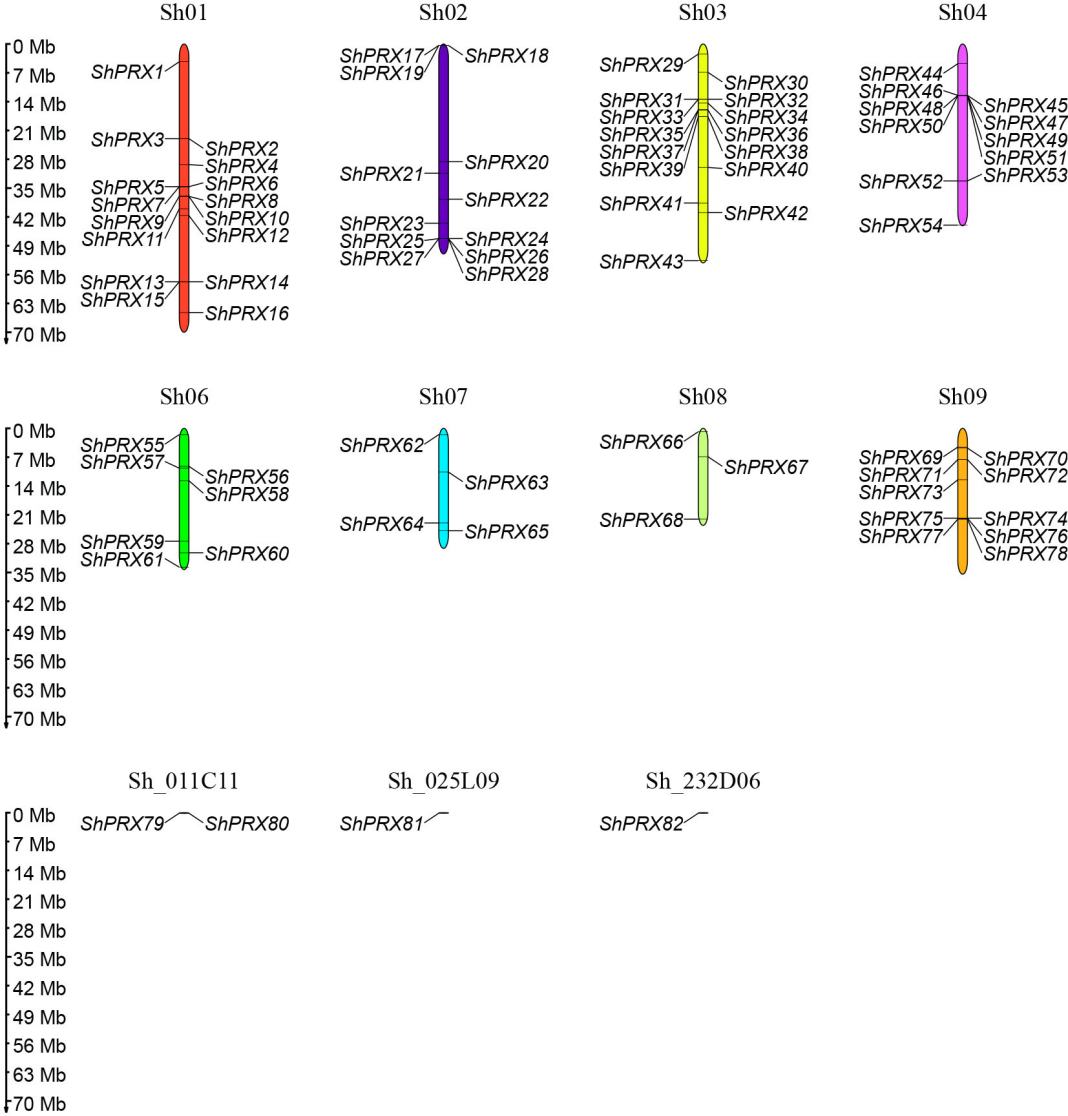
Distribution of the PRX gene family members on chromosomes in sugarcane.

The 82 *ShPRXs* were unevenly mapped onto the eight chromosomes of R570 STP (see **Figure 1**) based on the annotation information of the R570 STP genome. The *ShPRX* genes distributed in Sh01 (16), Sh02 (12), Sh03 (15), Sh04 (11), Sh06 (7), and Sh09 (10), while chromosomes Sh07 and Sh08 had only 4 and 3 *ShPRX* genes, respectively. Four *ShPRX* genes were distributed on scaffolds (Sh_011C11, Sh_025L09, and Sh_232D06).

### 3.2 Phylogenetic of the *PRX* gene family

**Figure 2:**
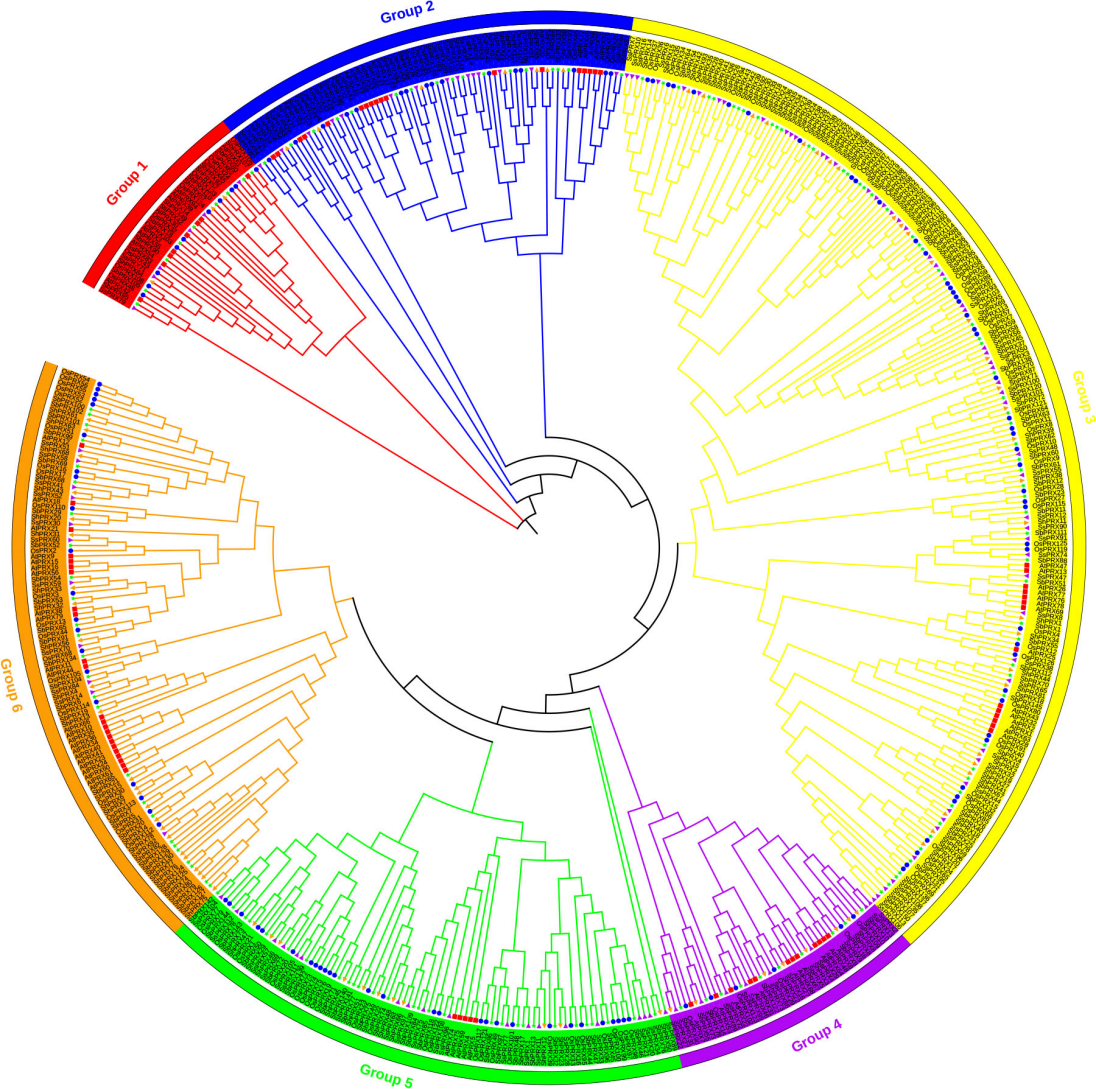
Phylogenetic analysis of PRX proteins from *A. thaliana, O. sativa, S. bicolor, S. spontaneum*, and *sugarcane. AtPRX*, red rectangle in the figure; *OsPRX*, blue circle in the figure; *SbPRX*, green star in the figure; *SsPRX*, purple right-pointing triangle in the figure; *ShPRX*, orange left-pointing triangle in the figure.

The phylogenetic tree from five plants was constructed on PRX amino acid to clarify the evolutionary relationship of the *PRX* gene family of sugarcane, as shown in **Figure 2**. The *ShPRX* family genes were divided into six groups. The phylogenetic tree based on the amino acid sequences of *ShPRX* indicated that the *ShPRX* family genes in groups 1, 2, 3, 4, 5, and 6 from five plants were clustered into groups I, II, VI, V, III and IV from sugarcane, besides *ShPRX1* (group 3), *ShPRX34* (group 3), *ShPRX22* (group 4), and *ShPRX62* (group 2). *ShPRX1* and *ShPRX34* were divided into group V, and *ShPRX22* and *ShPRX62* were divided into group IV (**Figure 2**; **Figure S1**). Group 1 comprised the lowest number of *PRX* genes. The *PRX* family genes of sugarcane shared high homology with the *PRX* family genes in sorghum and rice (**Figure 2**). The *ShPRX* family genes in other groups shared high homology to *S. spontaneum* or sorghum (**Figure 2**).

### 3.3 Gene structure and conserved motif analyses of the *ShPRX* gene family

**Figure 3:**
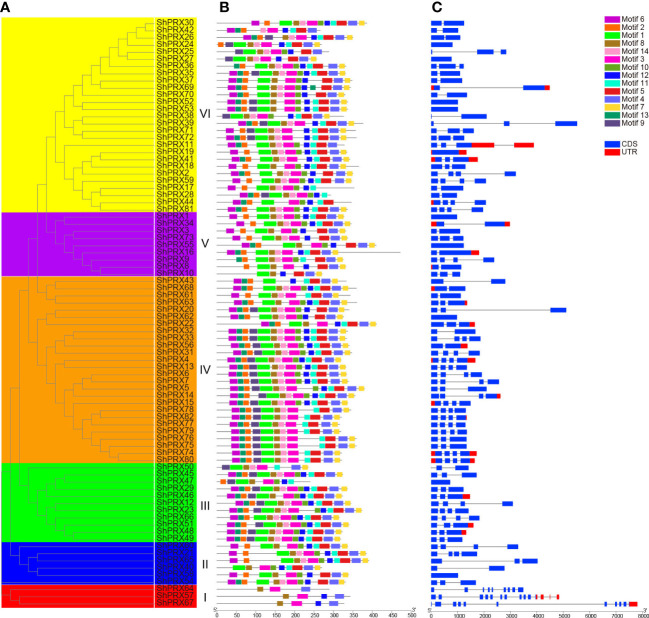
Analysis of gene structure and conserved motif of the PRX gene family in each group of sugarcane. **(A)** Phylogenetic tree of the *ShPRX* proteins. **(B)** Motif composition in the *ShPRX* proteins. **(C)** Gene structure of the *ShPRXs*.

The conservative structure of the *PRX* gene family was deciphered through the evolutionary relationship, motif, and structure of the PRX family genes in sugarcane (**Figure 3**). MEME was used to analyze the motif distribution within the *ShPRX* gene family, and 26 motifs were identified (p < 0.05) (**Table S3**). The *ShPRX* genes in the same group had the same motifs: the genes in Group I had motifs 3, 8, 18, and 19; Group II had motifs 3, 4, 5, 8, 10, and 12; Group III had motifs 3, 8, and 11; Group IV had motifs 2, 4, 5, 6, and 7; group V had motifs 1, 4, 7, and 8; and Group VI had motif 3, and most *ShPRX* genes have top 14 motifs (**Table S4**; **Figures 3A, B**). The *ShPRX* family genes had motif 3 except for *ShPRX10* and *ShPRX43*, and most genes had motif 8 except for *ShPRX43* and *ShPRX44* (**Figure 3B**).

According to the structural analysis of the *PRX* family genes, the length of these genes ranged from 723 to 7812 bp, where *ShPRX67* had the most considerable length and 11 coding regions, *ShPRX47* had the most petite length (**Table S1**; **Figure 3C**, **S2**). The respective structures of these genes were found to be similar in the same group, and 10 *PRX* genes had no introns. Most *PRX* genes contained 1–3 introns, but *ShPRX57*, *ShPRX64*, and *ShPRX67* in Group I contained 13, 8, and 10 introns, respectively, and *ShPRX20* (Group IV), *ShPRX39* (Group VI), and *ShPRX69* (Group VI) had long introns (**Figures 3A, C**).

### 3.4 *Cis*-acting elements and codon usage bias of *PRX* gene family in sugarcane

**Figure 4:**
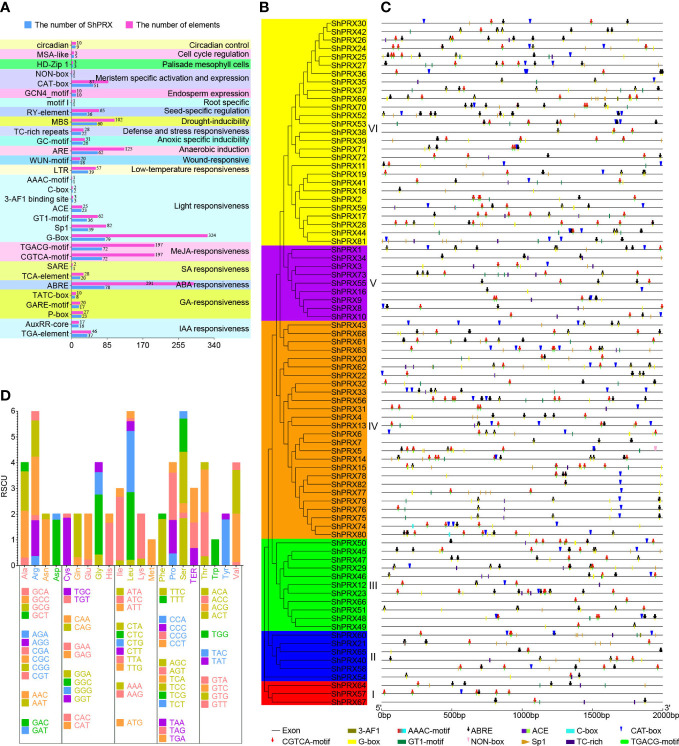
Analysis of cis-acting elements and codon usage bias of the PRX gene family in sugarcane. **(A)** The analysis of cis-acting elements of the PRX gene family in sugarcane. **(B)** The phylogenetic tree of the *ShPRX* proteins. **(C)** The analysis of cis-acting elements involved in the ABA, MeJA, light, defense, and stress responsiveness, meristem-specific activation, and expression in the PRX gene family of each group. **(D)** The codon usage bias analysis of PRX gene family in sugarcane.

*Cis*-acting elements can participate in gene expression and regulation, and members of the *ShPRX* gene family play a key role in biotic and abiotic stress. The function of the *ShPRX* family genes can be predicted by analyzing the *cis*-acting elements in the 2-kb upstream region of the *ShPRX* family genes. Thirty-one cis-acting elements were identified and involved in hormones, abiotic stress, tissue-specific cell cycle, and circadian control by analyzing and selecting *cis*-acting elements of the *ShPRX* family genes (**Table S5**). The differences were detected in the variety and number of *cis*-acting elements across the *ShPRX* family genes (**Table S5**). *ShPRX15*, *ShPRX41*, *ShPRX48*, and *ShPRX62* had the maximum variety of *cis*-acting elements (15), but the number of cis-acting elements in *ShPRX52* was the greatest (37). *ShPRX22*, *ShPRX38*, *ShPRX49*, and *ShPRX62* contained plant hormone-responsive elements, including IAA-, GA-, ABA-, SA-, and MeJA-responsive elements. We inferred that plant hormones might regulate these *ShPRX* family genes. *ShPRX31* and *ShPRX66* did not contain ABA and light-responsive elements but contained seed-specific regulation elements and MYB binding sites involved in drought inducibility (**Table S5**). *The cis*-acting regulatory elements of most *ShPRX* family genes were involved in ABA, MeJA, light responsiveness, anaerobic induction, and drought inducibility (**Figure 4A**).

The cis-acting elements in Groups III, IV, V, and VI, were involved in the meristem-specific activation and expression. Over half of those in the Group I, II, and V were involved in the defense and stress responsiveness (**Figures 4B, C**). The *cis*-acting regulatory elements in Group 1 involved in the IAA, ABA, SA, MeJA, light, low-temperature, and anaerobic induction, and group IV in the seed-specific regulation (**Table S6**). These results suggested that the *ShPRX* family genes might participate in the response of sugarcane to biotic and abiotic stresses and tissue-specific responses.

The codon usage bias and base composition analysis of coding sequences of the *PRX* family genes were calculated (**Table S7**, **S8**). The mean values of the codon composition at the third position from high to low were C3s (0.589), G3s (0.440), T3s (0.093), and A3s (0.080), and the mean content of the GC (65.1%) was also higher than AT (34.9%), suggesting a GC-rich composition of coding sequences of the *PRX* family genes (**Table S7**). The ENC values of coding sequences ranged from 28.25 to 58.62, with a mean of 38.059 (ENC < 40), and most ENC values of coding sequences were below 40, indicating a solid codon usage bias (**Table S7**). The RSCU revealed that 22 codons of 28 high-frequency codons (mean RSCU value > 1.0) were over-represented (mean RSCU value > 1.6). In comparison, 31 codons of 34 low-frequency codons (mean RSCU value < 1.0) were under-represented (mean RSCU value < 0.6) (**Figure 4D**; **Table S8**). The ENc plot (**Figure S3A**) and PR2 plot (**Figure S3B**) analyses showed that the codon use of the class III PRX family genes in sugarcane was affected by mutation and selection pressure.

### 3.5 Syntenic and selection pressure analysis of the *ShPRX* gene family

**Figure 5:**
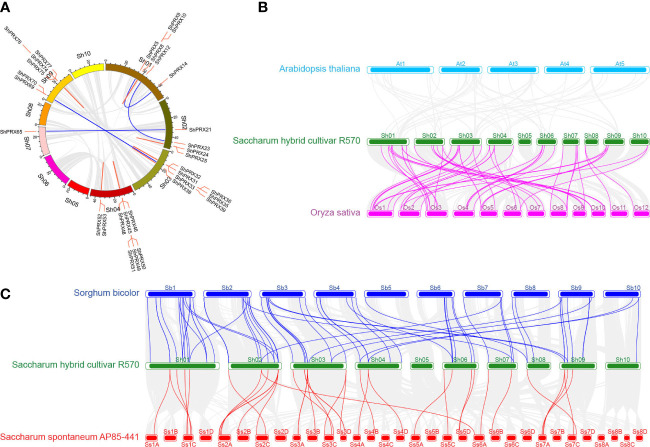
Syntenic analysis of PRX gene family in Sugarcane, *A. thaliana*, *O. sativa*, *S. bicolor*, and *S. spontaneum*. **(A)** Syntenic analysis of PRX gene family in sugarcane. Red lines represent tandem duplication PRX gene pairs, and blue lines represent segmental duplication PRX gene pairs. **(B)** Syntenic analysis of PRX gene family in Sugarcane, *A. thaliana*, and *O. sativa.* **(C)** Syntenic analysis of the PRX gene family in Sugarcane, *S. bicolor*, and *S. spontaneum*.

The syntenic relationships of the *ShPRXs* were analyzed to explore the genomic expansion of the *PRX* gene family in sugarcane. In total, 32 of the 82 *ShPRXs* had syntenic relationships, and 10 ones in five syntenic pairs underwent segmental duplication, while 22 genes in 13 syntenic pairs underwent tandem duplication (**Figure 5A**; **Table S9**). The Ka/Ks ratios of 17 of the 18 syntenic pairs were < 1, which might have undergone purifying selection, indicating that the evolution of these pairs was slow (**Figure 5A**; **Table S9**).

The syntenic relationships of the PRX family genes from *S officinarum*, *A. thaliana*, *O. sativa*, *S. bicolor*, and *S. spontaneum* were analyzed to better understand duplication and evolution of *PRX* gene. The results revealed that 52, 68, and 37 syntenic gene pairs of PRX genes were detected in *S officinarum versus O. sativa*, *S officinarum versus S. bicolor*, and *S officinarum versus S. spontaneum*, respectively (**Figures 5B, C**; **Table S9**). The Ka/Ks ratio of 1 of the 157 syntenic gene pairs was> 1, indicating that the syntenic gene pair had been strongly positively selected during evolutionary history (**Table S9**). The Ka/Ks ratios of 153 from the 157 syntenic gene pairs were < 1, which might have undergone purifying selection, indicating that the evolution of these pairs was slow. The duplication events of homologous collinearity gene pairs of the class III PRX occurred 4.094–85.36 Mya for sugarcane and rice, 21.992–83.6 Mya for sugarcane and sorghum, and 3.568–50.095 Mya for sugarcane and its wild relative of *S. spontaneum*. The class III PRX gene family has been identified in at least 29 plants in the whole genome. The duplication events occurred 159.9 Mya for 12 plants in monocotyledon from 21 plants in dicotyledon. Moreover, 104.7 Mya in *Poaceae* diverged from *Bromeliaceae* (**Figure S3C**; **Table S10**).

### 3.6 Expression profiles of the *PRX* family genes in different tissue

**Figure 6:**
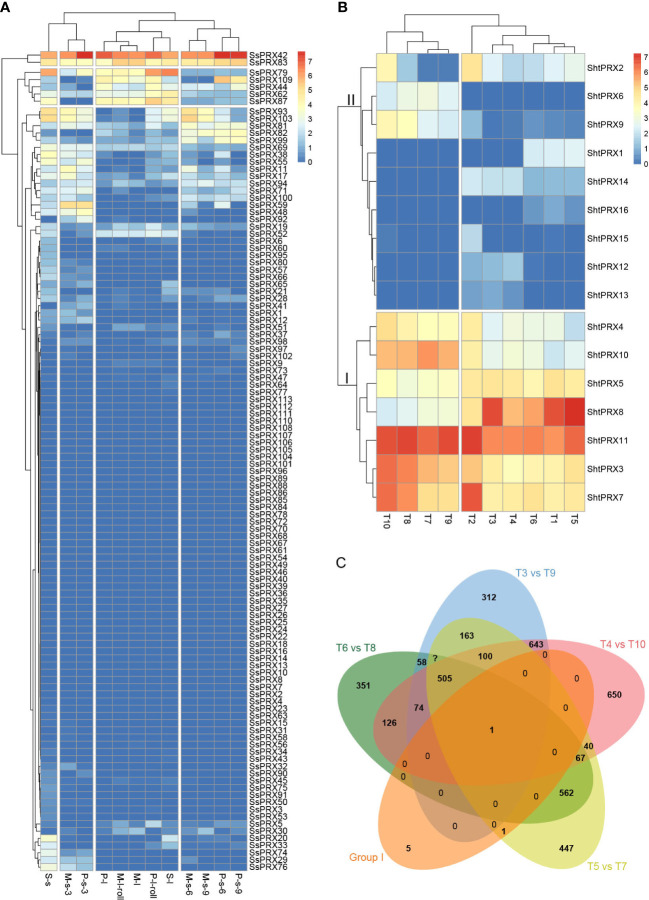
Expression pattern of the PRX genes in sugarcane. **(A)** Tissue-specific expression patterns of PRX gene family in *S. spontaneum*. S represents seedling; s represents stem; l represents leaf; P represents early maturity stage; M represents mature stage; 3, 6, and 9 represent sugarcane’s third, sixth, and ninth stem nodes. **(B)** Expression patterns of PRX gene family of sugarcane in response to SCMV infection. T1, the +1 leaf of Badila; T2, the −3 leaf of Badila; T3, the +1 leaf of virus-free Badila; T4, the −3 leaf of virus-free Badila; T5, the +1 leaf of B48; T6, the −3 leaf of B48; T7, the +1 leaf of B48 post-SCMV infection; T8, the −3 leaf of B48 post-SCMV infection, T9, the +1 leaf of virus-free Badila post-SCMV infection; T10, the −3 leaf of virus-free Badila post-SCMV infection. **(C)** The number of co-differentially expressed genes in groups I, T6_vs_T8, T3_vs_T9, T4_vs_T10, and T5_vs_T7.

Tissue-specific expression patterns of the *SsPRX* genes were analyzed based on the transcriptome data of *S. spontaneum* to explore the functions of the *PRX* gene family in sugarcane further. The 113 *SsPRX* genes were expressed as FPKM values in leaf roll, leaf, the third, sixth, and ninth stem nodes of sugarcane at the seedling, elongation and maturity stage (**Figure 6A**; **Table S11**). Four *SsPRX* genes (*SsPRX42*, *SsPRX62*, *SsPRX79*, and *SsPRX83*) were highly expressed in all tissues, and 69 *SsPRX* genes had a low expression or no expression, and the rest of the highly expressed *SsPRX* genes had tissue specificity.

### 3.7 Expression profiles of *PRX* family genes under SCMV, Cd, and salt stresses

Sixteen PRX proteins with a conserved PRX domain were characterized as members of the sugarcane class III *PRX* gene family based on the transcriptome data of Badila and B48 in response to SCMV infection. We named them *ShtPRX1*–*ShtPRX16* based on their Unigene IDs (**Table S2**). The expression patterns of the *ShtPRX* genes were analyzed on transcriptome data (**Table S12**). The *ShtPRX* genes were divided into two groups according to the clustering of expression patterns. Genes in Group I was primarily highly expressed in all samples. Genes in Group II, except for *ShtPRX13* and *ShtPRX16*, were primarily highly expressed in some samples (**Figure 6B**). ShtPRX8 was only one co-differentially expressed gene (|log_2_FC| ≥ 3, FDR < 0.05) in five groups (**Figure 6C**), indicating that *ShtPRX8* might participate in SCMV stress in sugarcane.

Homologous class III *PRX* genes of R570 were identified using class III PRX family protein sequences of rice as a library, and screened bu BLASTP at the e-value less than 1e^-5^. (**Table S13**). The expression profiles of rice under Cd and salt stresses were analyzed to infer the function of *PRX* homologous genes in sugarcane (**Tables S14**, **S15**; **Figure S4**). In groups #I, III, and VII, except for *OsPRX10*, the |log_2_FC| values for (Cd/CK)-WR or (Cd/CK)-WS was more than 3, and homologous genes, including *ShPRX38*, *ShBAC.PRX23*, *ShBAC.PRX41*, *ShBAC.PRX42*, *ShBAC.PRX43*, *ShBAC.PRX51*, *ShBAC.PRX52*, and *ShBAC.PRX75* might participate in Cd stress in sugarcane (**Figure S4A**; **Table S13**). In group 2, the |log_2_FC| of *OsPRX56* for PK-SS/PK-CK was more than 2, and homologous genes of *ShBAC.PRX20* might participate in salt stress in Sugarcane (**Figure S4B**; **Tables S13**, **S14**).

**Figure 7:**
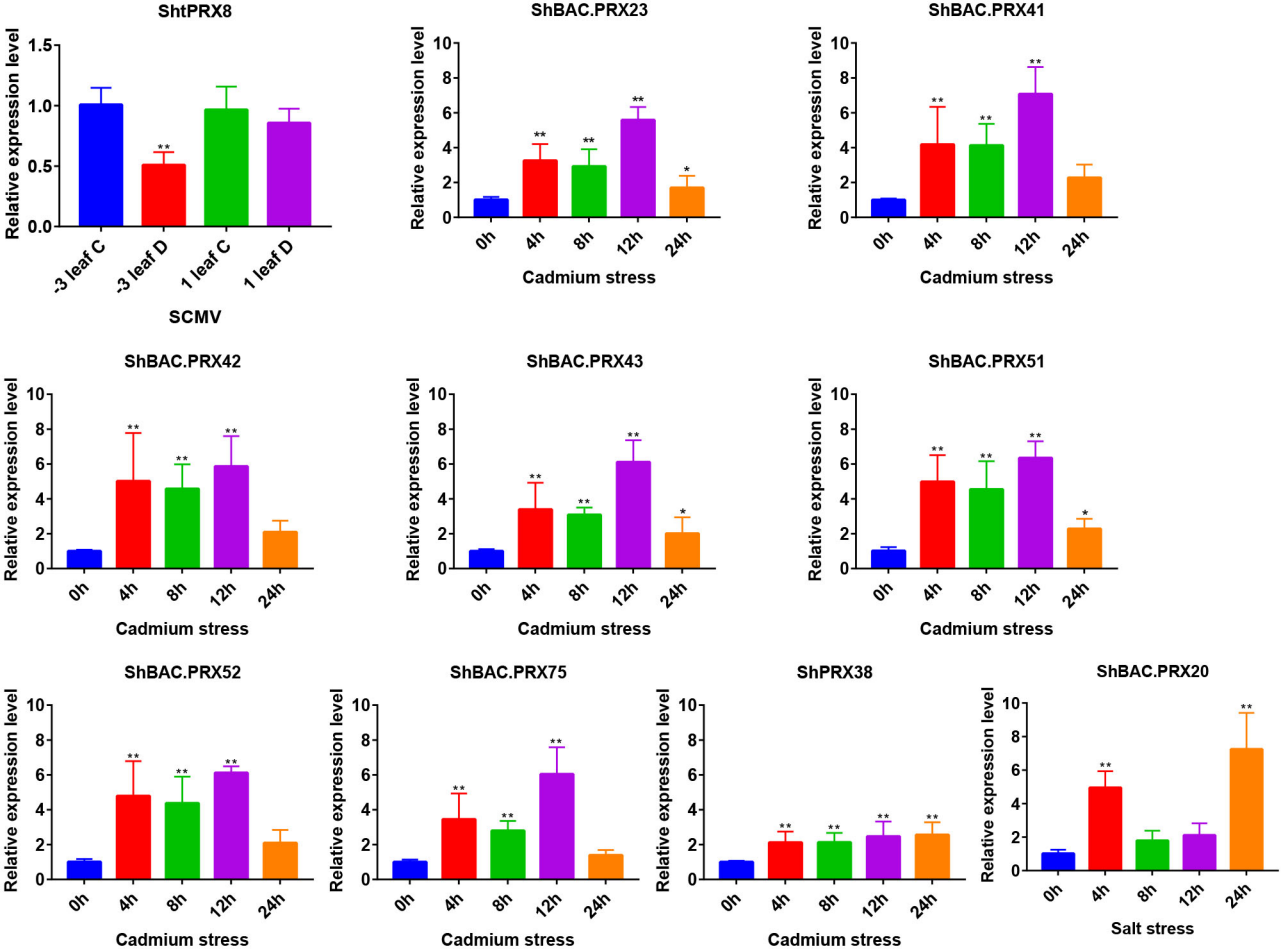
Analysis of SCMV, Cd, and salt stress differentially expressed PRX gene family members in sugarcane. The black bar graphs represent the relative expression levels of PRX family genes in the leaves under SCMV, Cd, and salt stresses. The −3 leaf C, the −3 leaf of virus-free Badila; the 1 leaf C, the +1 leaf of virus-free Badila; −3 leaf D, the -3 leaf of post-SCMV infection Badila (symptomatic leaf); 1 leaf D, the +1 leaf of post-SCMV infection Badila (asymptomatic leaf); *: 0.01 < p < 0.05, **: p < 0.01; The mean and SD were calculated from three biological and three technical replicate samples.

qRT-PCR analysis was performed to analyze the responses of the *PRX* family genes in sugarcane exposed to SCMV, Cd, and salt stresses. The primer sequences were listed in **Table S16**. The expression levels of *ShtPRX8* showed a significant decrease in Badila after SCMV infection. After applying 4.3 mM Cd^2+^ stress to sugarcane, the expression levels of *ShBAC.PRX23*, *ShBAC.PRX41*, *ShBAC.PRX42*, *ShBAC.PRX43*, *ShBAC.PRX51*, *ShBAC.PRX52*, *ShBAC.PRX*75 and *ShPRX38* showed a significant increase from 0 to 24 h (**Figure 7**). After applying 1% sodium chloride solution stress to sugarcane, the expression levels of *ShBAC PRX20* significantly increased from 0 to 24 h (**Figure 7**).

## 4 Discussion

Class III PRXs are essential in response to various biotic and abiotic stresses during plant growth and development. The class III *PRX* gene family has been identified in at least 29 plants in the whole genome, but the class III *PRX* gene family has yet to be studied in sugarcane. The number of *PRX* family genes identified in R570 BAC and STP was fewer than in other monocotyledons and dicotyledons. The class III *PRX* family genes were divided into six groups based on the phylogenetic analysis of sugarcane, sorghum, *S. spontaneum*, rice, and *A. thaliana* and the structure and conservative motif of class III PRX genes were similar in each group. The class III *PRX* genes in the same group were highly conserved. The identified class III *PRXs* in sugarcane had no collinear gene pair in *A. thaliana*. This is the same as Cesarino’s results, showing three sugarcane *PRXs*, being monocot-specific sequences, had no clear ortholog in *A. thaliana* (Cesarino et al., 2012).

*Poaceae* diverged from *Bromeliaceae* at 104.7 Mya. The duplication events of homologous collinearity gene pairs of the class III PRX for sugarcane and rice, sugarcane and sorghum, and sugarcane and its wild relative of *S. spontaneum* occurred at 0–85.36 Mya. These results revealed that class III *PRXs* in sugarcane was formed after *Poaceae* and *Bromeliaceae* diverged. Tandem duplication events play a leading role in the expansion of the class III *PRX* gene family in sugarcane, which was the same as *B. distachyon* (Zhu et al., 2019), foxtail millet (Ma et al., 2022), and grapevine (Xiao et al., 2020). Purifying selection essentially maintained the function of class III PRX proteins in sugarcane. However, tandem and segmental duplications are the primary reasons for expanding the class III *PRX* gene family in maize (Wang et al., 2015) and pineapple (Hou et al., 2022). Segmental duplication is the main reason for the expansion of the class III PRX gene family in tobacco (Cheng et al., 2022), soybean (Aleem et al., 2022), and potato (Yang et al., 2020). These results indicated that tandem and segmental duplications were pivotal in expanding plants’ class III *PRX* gene family.

Phylogenetic analysis revealed that *ShPRX* family genes shared high homology with the *PRX* family genes in *S. spontaneum* and sorghum, which was consistent with the genetic relationship between species. We inferred that *ShPRX* family genes might have the same function in the same group. GO enrichment analysis of motifs using InterProScan revealed that motifs 1, 2, 3, 4, 5, 18, 19, and 21 are essential in response to oxidative stress (GO:0006979) and have peroxidase activity (GO:0004601). However, there are some motifs with unknown functions in each group; these motifs might play a key role in the *ShPRX* family genes.

Gene expression patterns and cis-acting elements can provide important information regarding gene function. More than 87% of class III *ShPRX* genes were involved in light (G-box), ABA (ABRE), and MeJA (CGTCA and TGACG motifs) response, and more than 73% of class III PRX genes participated in anaerobic (ARE) and drought (MBS) responses. Also, over 62% of class III *PRX* genes participated in meristem-specific (CAT box) responses (**Table S5**). We inferred that class III *PRX* genes of sugarcane played an essential role in plant growth and development. The highest enzymatic activity was presented in the pith and rind of mature internodes at three different developmental stages of sugarcane (young, developing, and mature) (Cesarino et al., 2012). The expression of class III PRX proteins in susceptible sugarcane was inhibited for 72 h after *Sporisorium scitamineum* inoculation (Peters et al., 2017). Of 113 *SsPRX* genes, 69 exhibited little or no expression in tissues, indicating that *SsPRX* genes might be expressed under specific conditions or at specific developmental stages. Of 44 highly expressed genes, 26 and 43 were expressed in leaves and stems, respectively, and most of them might play an essential role in the leaf and stem of sugarcane.

In rice, the overexpression of *OsPrx30* contributed to maintaining a high level of PRX activity and reducing H_2_O_2_ content, thereby enhancing the rice plant’s susceptibility to *Xoo* (Liu et al., 2021), suggesting that class III *PRX* genes might have similar functions in sugarcane. The concentrations of H_2_O_2_ increased significantly in B48. The genes related to ROS-producing and scavenging pathways were differentially expressed on the ScMV-inoculated plants at days 3, 6, 9, and 12 (Akbar et al., 2020). The expression of *ShtPRX8* was significantly reduced post-SCMV inoculation based on the transcriptional data from our previous study. PRX, primarily existing in peroxisomes, could reduce the ROS level. SCMV could target intracellular peroxisomes for replication (Xie et al., 2021). The class III *PRX* family genes might play an essential role in sugarcane mosaic disease by activating the antioxidant system and regulating ROS and H_2_O_2_ content. Plants produced ROS under salt and Cd stresses, and the class III *PRX* family genes play an essential role in plant Cd and salt stress by scavenging ROS (Chiang et al., 2006; Kidwai et al., 2020; Su et al., 2020). The overexpression of the class III *PRX* family gene of *TaPRX-2A* in wheat activated the ABA pathway and antioxidant enzymes, leading to reduced ROS accumulation and increased osmotic metabolites, thereby enhancing salt tolerance (Su et al., 2020). The expression levels of class III PRXs significantly increased after applying 4.3 mM Cd and 1% NaCl solution stress to sugarcane. We inferred that the class III PRX family genes could enhance the tolerance of Cd and salt stresses in sugarcane by activating the antioxidant system and scavenging ROS.

## 5 Conclusion

Tandem duplication events play a leading role in the expansion of *ShPRX* genes, and purifying selection essentially maintains the function of *ShPRX* proteins. In this study, 82 *ShPRX* genes were identified in the R570 STP genome and divided them into six groups. Expression profile and qRT-PCR analyses showed that SCMV, Cd, and salt could specifically induce the expression of *PRX* genes of sugarcane. These results help understand the structure, evolution, and functions of the class III PRX gene family in sugarcane with a view of providing ideas for the phytoremediation of Cd-contaminated soil and breeding of new sugarcane varieties resistant to sugarcane mosaic disease, salt, and Cd in the future.

## Data availability statement

The datasets presented in this study can be found in online repositories. The names of the repository/repositories and accession number(s) can be found in the article/**Supplementary Material.**

## Author contributions

MZ, WY, and JX designed the research. HS wrote the draft manuscript. LF, LQ, HJ, ZD, HZ, and ZY performed the experiments and data analyses. GC and YB conducted the genomic analysis. JX and WY edited the first draft. MZ conceived the idea, provided supervision, revised the manuscript, and provided funds for this investigation. All authors contributed to the article and approved the submitted version.

## Supplementary material

Supplementary Figure 1 Phylogenetic analysis of ShPRX proteins.

Supplementary Figure 2 Analysis of gene structure and conserved motif of the PRX gene family.
