# Supplementary material for: Genome-wide identification of the class III peroxidase gene family of sugarcane and its expression profiles under stresses
Source: Front Plant Sci. 2023 Jan 30;14:1101665. doi: 10.3389/fpls.2023.1101665 (PMC9924293; doi:10.3389/fpls.2023.1101665)
Supplement: Supplementary Figure 1 — Phylogenetic analysis of ShPRX proteins. [file DataSheet_1.zip › Data Sheet20230105/Fig.S3 (A) ENc plot analysis of the class III PRX family genes in sugarcane. (B) PR2 plot analysis of class III PRX family genes in sugarcane. (C) Th.docx]

**
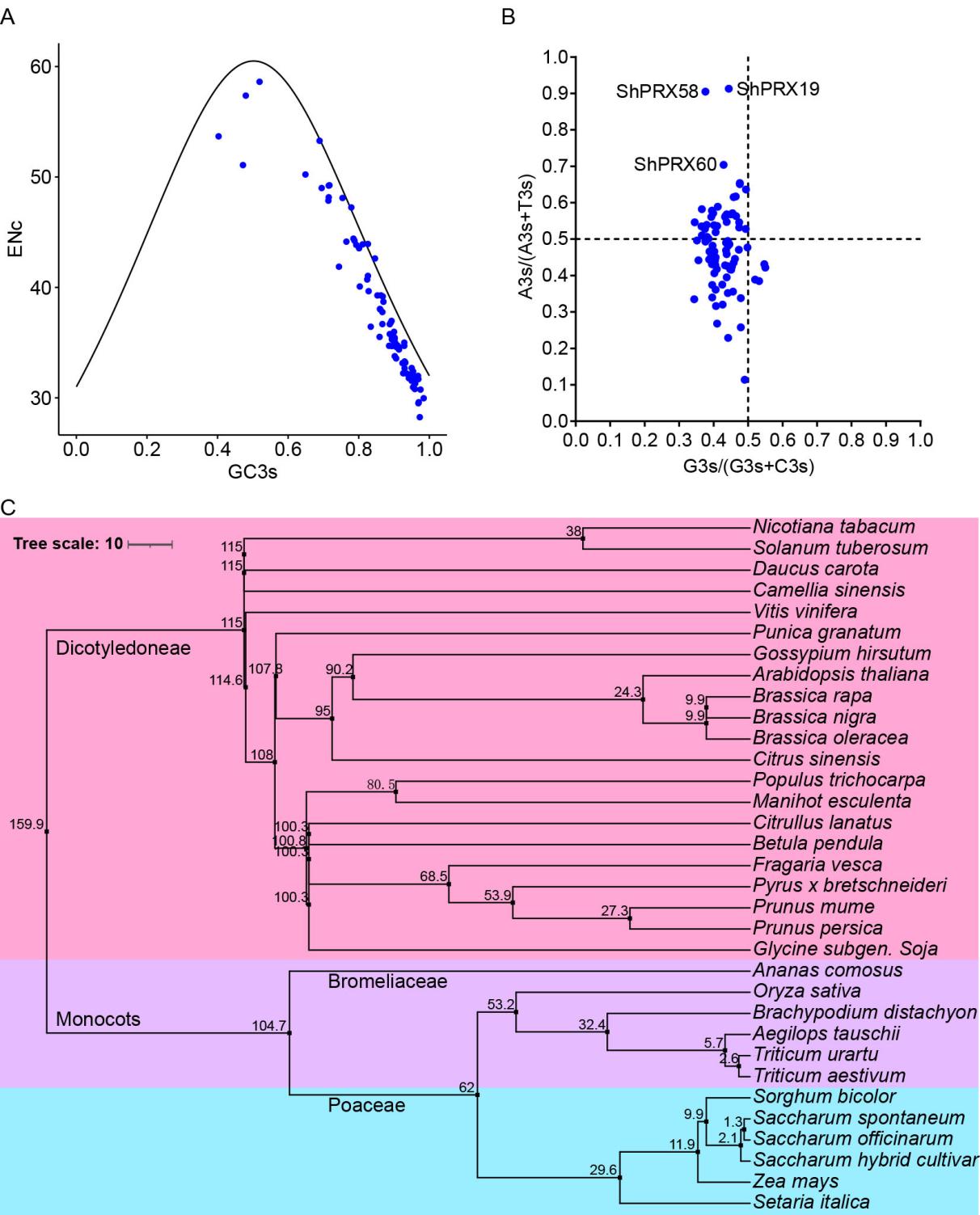
**

**Fig.S3 (A) ENc plot analysis of the class III PRX family genes in sugarcane.** **(B) PR2 plot analysis of class III PRX family genes in sugarcane. (C) The divergence time tree of *Saccharum hybrid cultivar.***
