# Supplementary material for: Genome-wide identification of the class III peroxidase gene family of sugarcane and its expression profiles under stresses
Source: Front Plant Sci. 2023 Jan 30;14:1101665. doi: 10.3389/fpls.2023.1101665 (PMC9924293; doi:10.3389/fpls.2023.1101665)
Supplement: Supplementary Figure 1 — Phylogenetic analysis of ShPRX proteins. [file DataSheet_1.zip › Data Sheet20230105/Fig.S4 Expression pattern of the PRX genes of rice in response to cadmium and salt stress.docx]

**
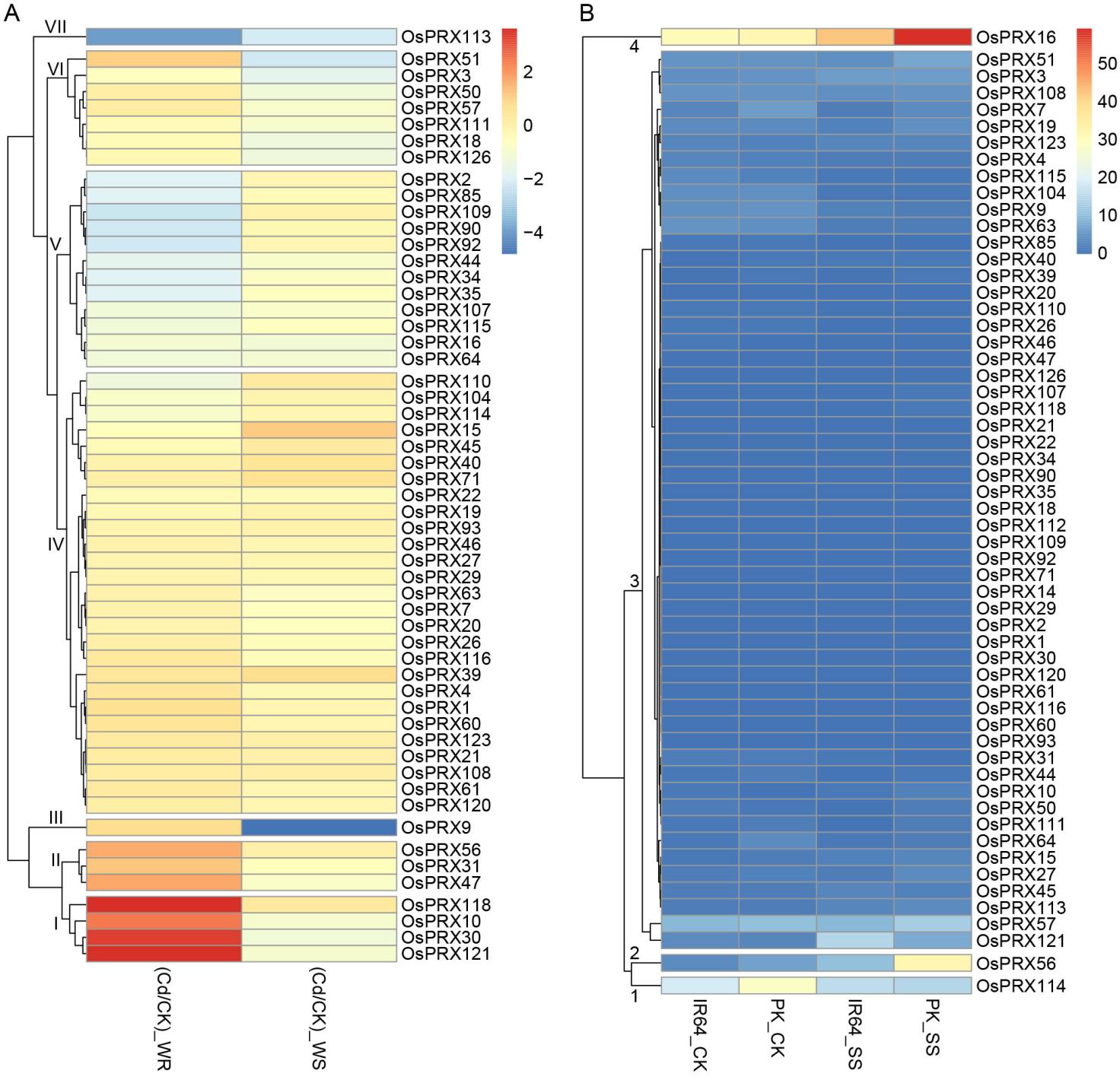
**

**Fig.S4 Expression pattern of the PRX genes of rice in response to cadmium and salt stress.**

**(A) Differential expression heatmaps of the PRX genes of rice in response to cadmium stress. Cd: under 10 μM CdCl_2_ added condition; CK: control condition; WS: the root of WT rice plants; WS: the shoot of WT rice plants. (B) Expression pattern of the PRX genes of rice in response to salt stress. IR64 and PK represents rice cultivars IR64 and Pokkali, respectively; CK represents control; SS represents salt stress.**
