# Supplementary figures and images for: Genome-wide identification of the class III peroxidase gene family of sugarcane and its expression profiles under stresses
Source: Front Plant Sci. 2023 Jan 30;14:1101665. doi: 10.3389/fpls.2023.1101665 (PMC9924293; doi:10.3389/fpls.2023.1101665)

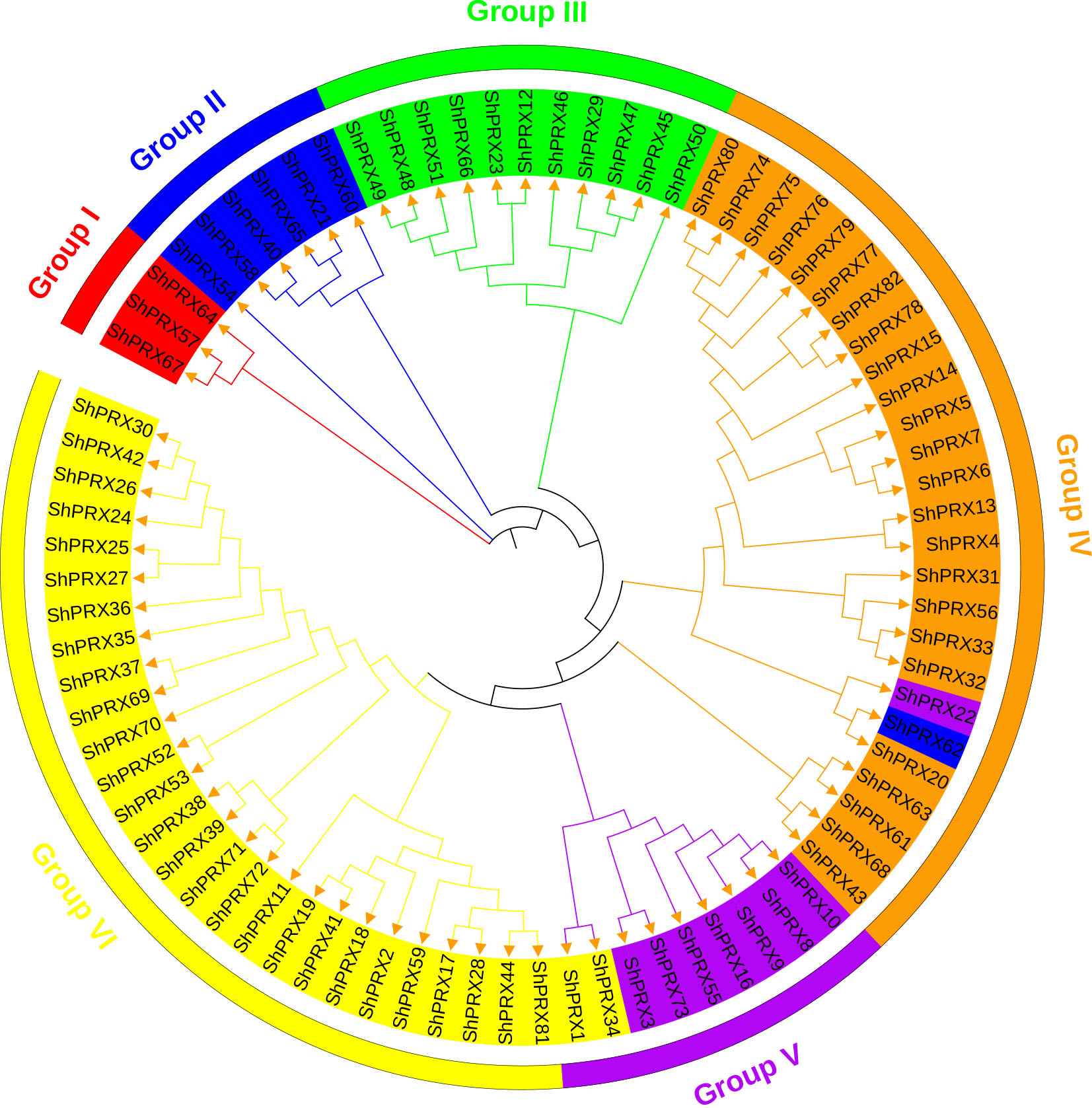

Supplement: Supplementary Figure 1 — Phylogenetic analysis of ShPRX proteins. [file DataSheet_1.zip › Data Sheet20230105/Fig.S1 Phylogenetic analysis of ShPRX proteins_300dpi.jpg]

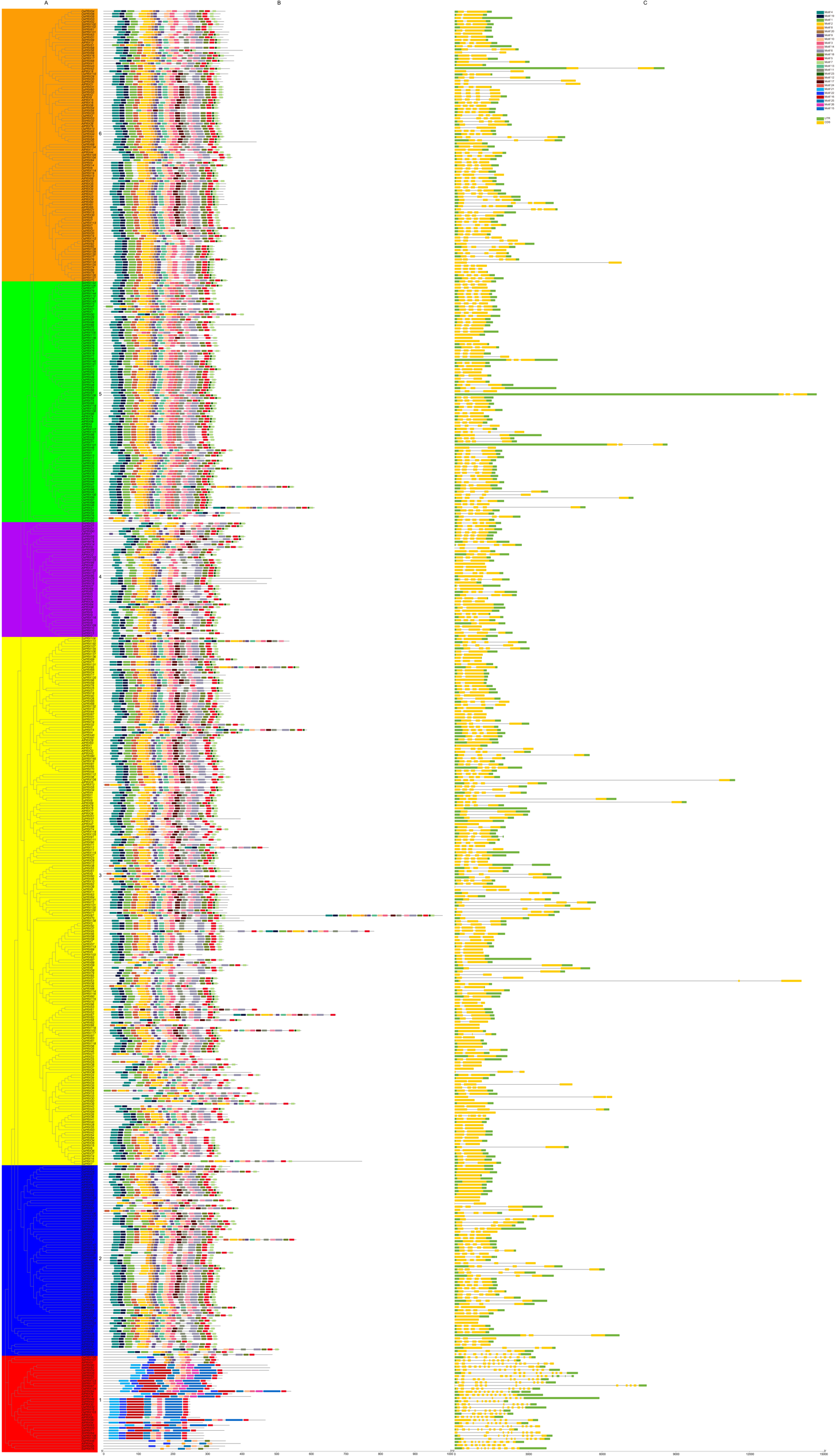

Supplement: Supplementary Figure 1 — Phylogenetic analysis of ShPRX proteins. [file DataSheet_1.zip › Data Sheet20230105/Fig.S2 Analysis of gene structure and conserved motif of the PRX gene family.pdf]
